# Buthionine sulfoximine increases the efficacy of arteether antimalarial activity in arteether-resistant *Plasmodium vinckei* by glutathione depletion

**DOI:** 10.5281/zenodo.10870048

**Published:** 2015-04-30

**Authors:** Ramesh Chandra, Santosh Kumar, Sunil Kumar Puri

**Affiliations:** 1 Division of Parasitology, Central Drug Research Institute, Lucknow 226001, India; ¶ Present address: Department of Anatomy and Neurobiology, School of Medicine, University of Maryland-Baltimore, Baltimore, MD 21201, USA

## Abstract

**Background:**

L-buthionine (S,R)-sulfoximine (BSO) regulates the glutathione (GSH) level, which in turn exhibits remarkable regulation of several important aspects of cellular metabolism. We hypothesised that increasing the cellular levels of glutathione leads to an increased resistance to arteether, whereas decreasing these by using a GSH inhibitor increases the parasite sensitivity to arteether in the rodent malaria parasite *Plasmodium vinckei*.

**Materials and Methods:**

We tested *in vivo* effects of BSO on GSH and hemozoin formation in arteether-sensitive and - resistant strains. Experimental groups of 7-8 Swiss mice were inoculated by intraperitoneal injection (i.p.) with 1×10^6^ parasitized erythrocytes of PvAS (sensitive) or PvAR (resistant) strain of *P. vinckei*. The infected mice were treated with BSO (Sigma) 400 mg/kg twice a day for four days and blood was collected after the last injection with BSO.

**Results:**

A relatively stronger inhibition of GSH level was observed in the blood of mice infected with resistant parasites (62.64%; p<0.0001), whereas inhibition in sensitive strain-infected mice and uninfected mice was 32% (p=0.034) and 35% (p=0.034), respectively. The results also show an inverse relationship between GSH and hemozoin in the arteether-sensitive and -resistant strains. The hemozoin contents in the resistant strain are 0.27±0.09, 0.69±0.14 and 5.30±0.79 μmol/10^9^ cells at 5, 10 and 20% parasitemia, respectively, whereas hemozoin contents in the sensitive strain at the same parasitemia levels are 0.59±0.29, 12.38±1.96 and 30.80±2.27 μmol/10^9^ cells. Moreover, hemozoin formation increased by 80% through the administration of BSO in the arteether-resistant strain, whereas insignificant changes occurred in the sensitive strain. BSO was also found to increase the efficacy of arteether antimalarial activity against the resistant strain *in vivo*.

**Conclusions:**

Treatment with BSO significantly reduces the level of GSH, which leads to insufficient growth of resistant parasites. These results suggest that BSO might be helpful in prolonging the persistence of the drug, and pose a promising lead to help reducing the chance of resistance development against artemisinin and its derivatives.

## 1 Introduction

Malaria remains one of the most prevalent infectious diseases in the world, with 3.4 billion humans currently at risk [1]. The emergence of resistance to artemisinin is an urgent public health concern. Artemisinin and its derivatives are the most effective antimalarial drugs that act against chloroquine-resistant *Plasmodium falciparum* [2]. The mode of action and mechanism of resistance to artemisinin are still debated [3,4]. An artemisinin-resistant strain of *P. yoelii* is characterised by higher levels of translationally controlled tumour protein (TCTP) than sensitive strains; TCTP has a role in alkylation of heme and parasite proteins [5]. It has been suggested that resistance in *P. falciparum* isolates could become apparent via mutations in *PfATP6*, which is a membrane protein involved in cellular Ca^2+^ homoeostasis [6,7]. Introduction of the mutation L263E in the *PfATP6* gene exhibited a notable decrease in response to artemisinin and dihydroartemisinin [8]. This mechanism of action is further suggested by the activation of the endoperoxide bridge, which is a prerequisite for the generation of ROS and carbon-centred radicals and, hence, subsequent alkylation of essential parasite proteins [9]. During the erythrocytic stages, toxic free heme is released from the host haemoglobin and converted into non-toxic hemozoin [10]. The cytoplasmic free heme is also degraded by glutathione (GSH) in the cytosol [11]. Intracellular GSH also plays a role in protecting the cell against oxidative stress [12,13]. GSH is synthesised by the step-limiting enzyme γ-glutamyl cysteine synthetase (γ-GCS) and glutathione synthetase. Resistance in *Plasmodium* and cancer cells are accompanied by an increase in glutathione levels through higher expression of γ-GCS and glutathione reductase [14,15]. These are promising targets for the development of novel antimalarial agents [14,16]. The antimalarial drug methylene blue has a synergistic effect with artemisinin by inhibiting glutathione reductase [17]. GSH also alters the sensitivity of chloroquine-resistant *P. falciparum* Dd2 [18]. L-buthionine (S,R)-sulfoximine (BSO) is a specific inhibitor of γ-GCS [19,20], which significantly increases the sensitivity to chloroquine in resistant *P. berghei* and *P. falciparum* [18,21]. BSO inhibits the growth of *P. falciparum, P. berghei*, *Trypanosoma brucei* and *T. cruzi* [14,22-24] by altering the glutathione levels. BSO has been shown to reduce the sensitivity of artesunate in resistant cancer cells [25,26]. Acetaminophen, indomethacin and disulfiram were able to potentiate the antimalarial action of chloroquine and amodiaquine in *P. berghei*- or *P. vinckei petteri*-infected mice by an indirect decrease in the level of GSH [27]. Amodiaquine treatment failure was also associated with glutathione in *P. falciparum-*infected patients in Colombia [28]. These results are consistent with the expectation that the combination of drugs that can deplete parasite GSH *in vivo* could potentiate the antimalarial action of arteether. Artemisinin-based combinatory therapies (ACTs) have been introduced and are widely deployed in malarious regions to reduce the chance of resistance [29]. The WHO has also recommended a policy of ACTs for treatment for *P. falciparum.* Studying the mechanisms of resistance and efficacy may be helpful in prolonging the useful lifespan of the drug. In this study we report the *in vivo* efficacy of arteether in combination with BSO against an arteether-resistant strain of *P. vinckei*. In addition, we determined the effect of BSO on hemozoin formation for mechanistic purposes, which is a known target of the antimalarial action of several drugs against intraerythrocytic asexual stages [30].

## 2 Materials and Methods

### 2.1 Mice and parasites

Swiss albino mice, weighing 24-26 g, were obtained from the Division of Laboratory Animals, Central Drug Research Institute, Lucknow, India. The mice were sheltered in the animal facility at the institute and maintained on a commercial pellet diet and water *ad libitum* under standard housing conditions. Ethical guidelines on handling, care and use of experimental animals were followed during the conduct of the study [31]. The stable selected arteether-resistant rodent malaria parasite (PvAR) was maintained by drug pressure, which showed >24- fold resistance at the time of study [32]. However, inoculum for each experiment was obtained from infected mice left untreated for one passage. The *P. vinckei* arteether-sensitive strain (PvAS) was maintained in the laboratory by routine weekly blood passage.

### 2.2 Effect of BSO on glutathione

Experimental groups of 7-8 Swiss mice were inoculated by intraperitoneal injection (i.p.) with 1×10^6^ parasitized erythrocytes of the PvAS or PvAR strain. The infected mice were treated with BSO (Sigma) 400 mg/kg twice a day for four days (i.p.) and blood was collected after the last injection of BSO as described before [22]. The white blood cells and platelets were removed by using a Whatman CF11 cellulose column [33]. Blood was washed with cold PBS and then resuspended in 50 mM phosphate buffer (pH 6.5). The free parasite was isolated by treatment with saponin (0.15%) for 10 min at 4°C. Parasites or blood lysates were sonicated in 50 mM phosphate buffer (pH 6.5) containing the protease inhibitor cocktail. The resultant supernatants were then treated with equal volume of 5% 5-sulfosalicylic acid (SSA) solution (Sigma-Aldrich) to precipitate the protein. The mixture was allowed to stand at 4° C for 15 min and centrifuged at 3000 g for 5 min. The volume of the supernatant was used for the measurement of total glutathione by using the Brehe and Burch method [34].

### 2.3 Isolation of hemozoin

The hemozoin formation was measured at lower (5%), medium (10%) and higher (20%) parasitemia in PvAS- or PvAR-infected mice. The effect of BSO on hemozoin formation was also studied, and samples were prepared as described above. Hemozoin was isolated from infected blood by using the method by Coban *et al.* [35], and an extinction coefficient of 91 mM^−1^ cm^−1^ was used for quantification [36].

### 2.4 In vivo effect of BSO in combination with arteether

Two sets of experiments were conducted for this study. Groups of six mice were inoculated with 1×10^6^ PvAR-infected erythrocytes. The first group of mice was treated with BSO twice a day for four days at a dose of 200 or 400 mg/kg, alone or combined with arteether (5 mg/kg/day). The groups of mice in the second experiment were treated with the same dose of BSO but with a higher dose of arteether (10 mg/kg). The combined effect of parasitemia was compared with the group of mice treated with arteether alone. Mice in the control group received drug-free groundnut oil. Blood smears were prepared for each mouse every alternate day, fixed in methanol and stained with Giemsa for 30 min. The slides were washed in tap water, dried and examined in at least 20 fields per slide using 100× magnification microscopy to determine the percentage of infected erythrocytes.

### 2.5 Statistical analysis

The GSH level, hemozoin formation and parasitemia were compared using Student's *t*-test. A probability level of p<0.05 was considered statistically significant. Statistical analysis and graph work were performed using GraphPad Prism 3.03 (GraphPad Software).

## 3 Results

### 3.1 Effect of BSO on GSH level in infected blood and isolated parasites

**Figure 1.**
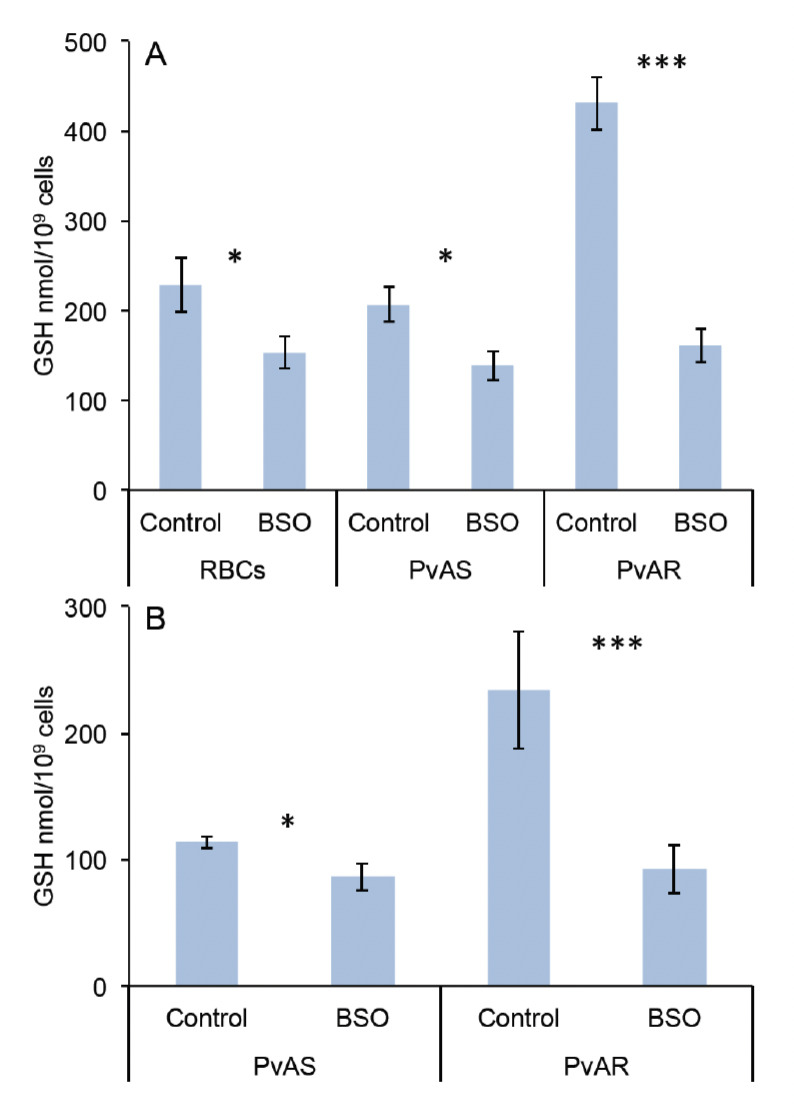
GSH levels in PvAS- or PvAR-infected and uninfected blood (A) and in isolated parasites (B) with and without BSO treatment. The s.d. is indicated for each point obtained from three biological replicates; *; *** indicate significant differences at p<0.05 and p<0.0001, respectively.

We previously reported that the PvAR had higher GSH levels in arteether-resistant *P. vinckei* [18]. To test the effect of BSO on GSH level in *in vivo*, PvAR- or PvAS-infected mice were treated with BSO followed by collection of blood at 15-20% parasitemia. The results show that BSO significantly reduced (62.64%; p=0.0001) GSH in PvAR-infected mice from 431±30 to 161±20 nmol/10^9^ cells, although this treatment was less effective on PvAS-infected (32%; p=0.034) and uninfected mice (35%; p=0.034; Figure 1a). The effect of BSO was also confirmed in isolated parasites, and the results show that it suppressed 60% GSH level (p=0.0001) from 273±20 to 92±19 nmol/10^9^ cells in PvAR parasites (Figure 1b). However, reduction of GSH in sensitive parasites was around 25% (p=0.029). These results indicate that the higher depletion of GSH level might be attributable to higher relative abundance in PvAR parasites.

### 3.2 Hemozoin formation and effect of BSO

**Figure 2.**
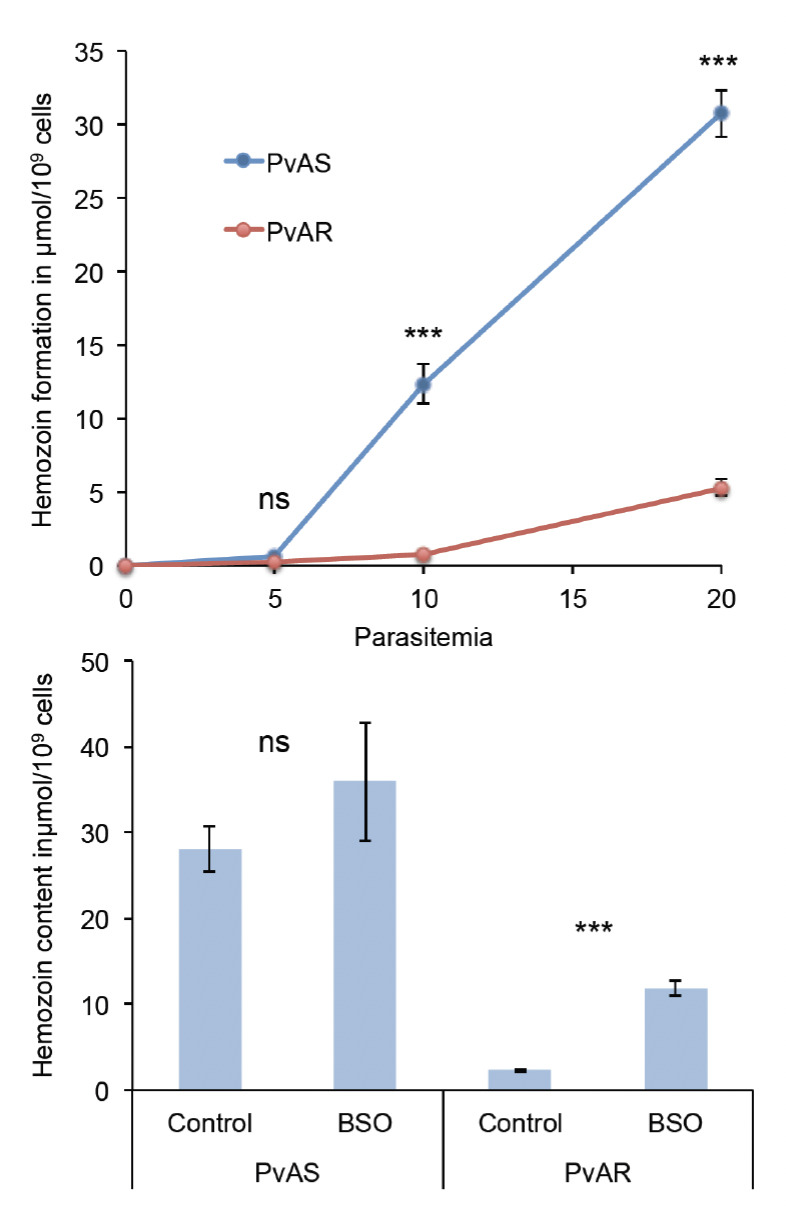
Trends of hemozoin formation in PvAS/PvAR parasites at 5, 10 and 20% parasitemia (A). Effect of BSO on hemozoin formation in treated and untreated mice (B). The s.d. is indicated for each point obtained from three biological replicates; *** indicates significant difference p<0.0001; ns: not significant.

**Figure 3.**
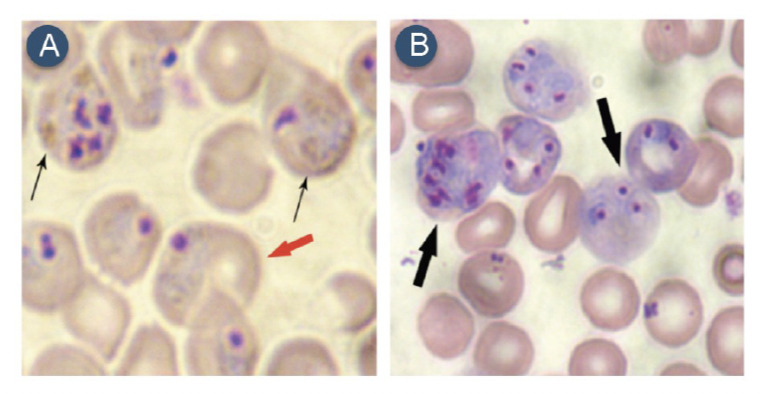
Giemsa-stained blood smears from PvAS- (A) and PvAR (B)-infected parasites. Cells represented by infected normocytes (A, red arrow), infected reticulocytes (B, black arrows). Figure A also shows hemozoin pigments (dark granules) in the PvAS parasite (thin arrows), which is not visible in the PvAR parasite.

As GSH has been postulated to play a role in the disposal of heme [11], studies were conducted to estimate hemozoin formation in arteether-sensitive and -resistant parasites. The hemozoin formation was determined at lower (5%), medium (10%) and higher (20%) parasitemia. The results showed that hemozoin contents in PvAR parasites were dramatically reduced to 0.27±0.10, 0.69±0.10 and 5±0.80 μmol/10^9^ cells at 5, 10 and 20% parasitemia, respectively, whereas hemozoin contents in PvAS parasites under the same parasitemia levels were 0.59±0.30, 12±20 and 30±20 μmol/10^9^ cells, respectively (Figure 2a). Thus, the results show that PvAR parasites considerably reduce the ability to convert free heme into hemozoin. The microscopic examination suggests that malaria pigments are not visible in PvAR parasites, while it was visible in sensitive parasites in the form of dark granules (Figure 3a,b). To test the *in vivo* effect of BSO on hemozoin formation, hemozoin was isolated from BSO-treated PvAR- or PvAS-infected blood. A significantly increased (p=0.0008) hemozoin formation to the extent of 80% was found in PvAR-infected mice, and the mean values of hemozoin levels in BSO-treated, and untreated parasite were 3±0.40 and 11±0.90 μmol/10^9^ cells, respectively (Figure 2b). PvAS-infected mice showed little change in hemozoin formation from 28±4.5 to 35±11.8 μmol/10^9^ cells (p=0.34).

### 3.3 Resistance reversal by BSO

**Figure 4.**
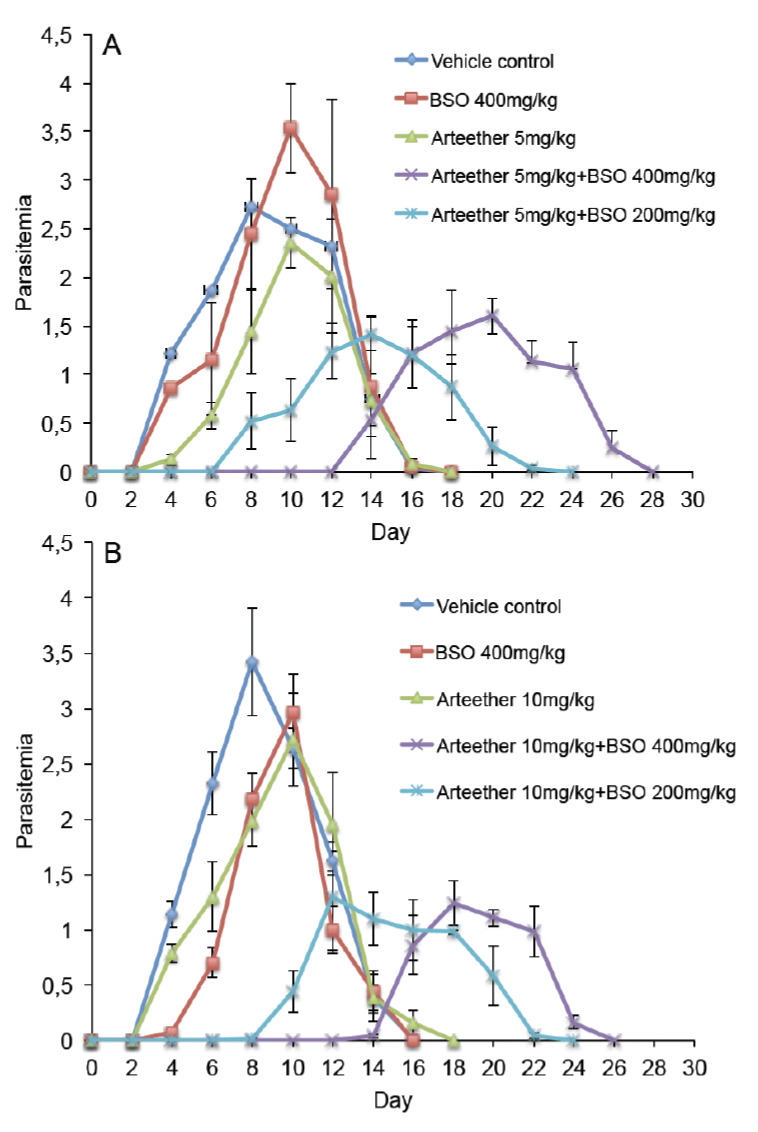
Parasitemia of PvAR-infected mice treated with 200 or 400 mg/kg BSO and/or 5 mg/kg arteether (A) and/or 10 mg/kg arteether (B). Injections were given on days 0-3 post-inoculation. Parasitemia was calculated by counting the number of parasite-infected erythrocytes per 1000 total erythrocytes. Results are presented as arithmetic means of six mice per group. Average values±s.e.m. are shown and the experiment was repeated twice with similar results.

To see the *in vivo* effect of BSO in combination with arteether on PvAR parasite proliferation, mice were treated on days 0-3 post infection and the parasitemia was measured on alternate days. The results show that a delay in the appearance of parasitemia was observed as a result of combination therapy when compared with arteether alone. Figure 4a shows that the combined doses (BSO 200 mg/ kg+5 mg/kg) prevented the appearance of parasitemia by approximately one week, although 3 and 5 mice became patent on days 8 and 10 p.i., respectively. This treatment also significantly reduced peak parasitemia when compared to arteether (p=0.030), BSO only treated (p=0.003) and untreated mice (p=0.020). Further delay in the appearance of parasitemia was observed on day 12 (3/6, 4/6 and 6/6 on day 12, 14 and 16, respectively) at high BSO dose (400 mg/kg). This treatment also significantly reduced the peak parasitemia when compared with arteether treated (p=0.046), BSO only treated (p=0.004), and untreated mice (p=0.027; Figure 4a).

We further examined the efficacy of BSO at a higher dose of arteether (10 mg/kg). No parasitemia was detectable up to day 6, and 3, 4 and 5 mice became patent on days 8, 10 and 12, respectively. Moreover, the peak parasitemia in the combination group was significantly less when compared to arteether (p=0.024), BSO (p=0.011) and untreated mice (p=0.002) at a dose of 200 mg/kg BSO+10 mg/kg arteether (Figure 4b). A high dose (400 mg/kg BSO+10 mg/kg arteether) further increased the delay, which was observed on day 12 (2/6, 3/6 and 6/6 on days 12, 14, and 16, respectively). These treatments also significantly reduced peak parasitemia when compared with arteether (p=0.039), BSO (p=0.002) and untreated mice (p=0.0009; Figure 4b). In this experiment BSO significantly reduced the parasitemia on days 6 and 8. All control groups of mice (arteether, BSO-treated and untreated mice) became positive on day 4. These results showed that BSO enhanced the efficacy of arteether against resistant parasites, thereby exhibiting potential for resistance reversal. The PvAR infection was not lethal to the mice, and all treated and untreated mice survived until the end of the experiment [37]. Treatment of the infected mice with arteether alone non-significantly reduced the parasitemia caused by the resistant strain of *P. vinckei*. In all cases, BSO treatment alone had no adverse effect or obvious toxicity on experimental animals, and BSO did also not significantly affect antimalarial activity (Figure 4a,b).

## 4 Discussion

Our group has previously reported that higher arteether drug pressure correlates with a significant increase in parasite GSH levels [32]. We further hypothesised that inhibition of GSH levels in arteether-resistant *P. vinckei* through administration of a GSH inhibitor increases parasite sensitivity to arteether. BSO is used in chemotherapy, in which it reduces levels of glutathione and increases the sensitivity of drugs [18,21,25,26]. We observed a significant reduction of GSH levels in the PvAR strain after the treatment with BSO as compared with the PvAS strain. It was depleted around 60% in PvAR-infected blood, compared with 32% and 35% in PvAS-infected and uninfected blood, respectively. The depletion of GSH depends upon BSO concentration used in *P. berghei*- and *P. falciparum*-infected and uninfected blood [18,22]. The present studies have demonstrated that hemozoin formation in PvAR parasite is dramatically reduced when compared with PvAS parasites. The visual appearance of the parasite also strongly supports this evidence (Fig. 3A,B). A reduction of hemozoin contents in chloroquine-resistant parasites has been reported for *P. berghei* and *P. falciparum* [38,39]. Our results also show that the arteether-resistant parasites have a preference for invading reticulocytes, which have higher GSH levels. This might lead to more efficient GSH-mediated heme detoxification in reticulocytes [40]. It has therefore been hypothesised that GSH might detoxify heme, thus explaining the decrease in hemozoin production. We have further shown that the malaria pigment reappears when treated with BSO in the PvAR strain. The transient inhibition of GSH by BSO treatment leads to a strong increase in the hemozoin production in chloroquine -resistant *P. berghei* [22].

We also evaluated that arteether is more effective at a lower dose in combination with BSO when compared with arteether treatment only. BSO alone does not have significant antimalarial activity at a lower dose (200 mg/kg). However, a significant inhibition of parasitemia was observed at the beginning of infection (day 4-6) at the higher dose (400 mg/kg), which later became non-significant (Fig. 4A,B). These results are in contrast with previously reported *P. berghei* and *P. falciparum* growth [14, 22]. The doses of BSO were more effective than the increased dose of arteether tested (Fig. 4A,B). This indicates that the pronounced oxidative stress might contribute to these events. Results have also emphasised that BSO sensitized the PvAR parasite at lower doses (5 mg/kg) of arteether, although the PvAR strain has shown resistance against 60 mg/kg arteether or higher, whereas the curative dose of PvAS strain is 2.5 mg/kg [32,41]. This phenomenon would deprive *Plasmodium* from GSH that normally protects it against increased oxidant stress and might alter the related enzyme activities. BSO enhances the susceptibility to chloroquine in *P. berghei* parasites by alteration of GSH levels [21,27]. The sensitivity of chloroquine in resistant *P. berghei* and *P. falciparum* parasite was increased after administration of BSO by alteration in GSH levels [42,43]. Antiretroviral protease inhibitors are able to enhance the sensitivity of chloroquine-resistant malaria parasites to the antimalarial drug by influencing the levels of GSH and the activities of the related enzymes [44].

The alteration of GSH might not be associated with mechanisms of arteether resistance, but is shown to be involved in the classical antioxidant defence. However, glutathione is also involved in the resistance against chloroquine and amodiaquine. Moreover, resistance against artemisinin is known to be multigenic and multifactorial. In conclusion, the results of this study demonstrate that BSO can be used to increase the efficacy of the antimalarial action of arteether *in vivo*. The combination of arteether with BSO could serve to treat malaria patients infected with artemisinin-resistant strains.

## 5 Conclusions

Arteether-resistant parasites contain higher GSH levels and less hemozoin than susceptible parasites. Treatment of BSO in combination of arteether increases the parasitemia in resistant parasite-infected mice. It might therefore be useful in extending the half-life of the drug and poses a promising lead to help reducing the chance of resistance development against artemisinin and its derivatives.
